# Gut microbiota–neuroimmune crosstalk in autoimmune encephalitis: mechanistic insights and therapeutic prospects

**DOI:** 10.3389/fmicb.2026.1946719

**Published:** 2026-09-14

**Authors:** Haiyan Zhang, Xue Zhou, Yugang Ma, Shoujian Zong, Xinqi Liu, Zongzhou Liu, Xingchen Wang

**Affiliations:** 1 The First Clinical Medical College, Shandong University of Traditional Chinese Medicine, Jinan, China; 2 Department of Neurology, Second Affiliated Hospital of Shandong University of Traditional Chinese Medicine, Jinan, Shandong, China; 3 Affiliated Hospital of Shandong University of Traditional Chinese Medicine, Jinan, China; 4 The Second Clinical Medical College, Shandong University of Traditional Chinese Medicine, Jinan, China; 5 Department of Rehabilitation, Guang'anmen Hospital Jinan Hospital (Jinan Hospital of Traditional Chinese Medicine), Jinan, China; 6 Department of Traditional Chinese Medicine, Shandong Rehabilitation Hospital, Jinan, China

**Keywords:** autoimmune encephalitis, gut microbiota, gut–brain axis, microbiota-based therapy, neuroimmune interactions

## Abstract

Autoimmune encephalitis (AE) comprises a heterogeneous group of inflammatory disorders of the central nervous system mediated by autoimmune responses, involving multiple pathological processes, including autoantibody production, aberrant immune-cell activation, and persistent neuroinflammation. In recent years, accumulating evidence has suggested that the gut microbiota may represent an important regulatory interface linking host metabolism, immunity, and the nervous system. Through microbial structural components, metabolites, and secreted signals, the gut microbiota may influence peripheral immune responses, blood–brain barrier integrity, and central nervous system inflammation. Conversely, neuroimmune dysregulation may reshape the intestinal microbial environment through neuroendocrine–immune regulatory networks, thereby establishing a dynamic bidirectional relationship between the gut microbiota and the neuroimmune system. However, the mechanisms underlying gut microbiota–neuroimmune interactions in AE, particularly their potential changes across the disease course, remain incompletely understood. This review summarizes current evidence regarding AE-associated alterations in the gut microbiota and disturbances of neuroimmune homeostasis, with particular emphasis on the potential molecular mechanisms through which the gut microbiota modulates neuroimmune responses and, reciprocally, the neuroimmune system reshapes the intestinal microbial environment. Within a proposed phase-based conceptual framework encompassing disease initiation, progression, and the chronic phase, we further discuss the potential dynamic changes in gut microbiota–neuroimmune crosstalk during AE. In addition, we summarize emerging therapeutic strategies, including dietary and nutritional interventions, probiotics, modulation of microbial metabolic functions, fecal microbiota transplantation, and precision microbiome engineering, and discuss their current evidence base and translational potential. Collectively, gut microbiota–neuroimmune crosstalk provides a useful conceptual framework for understanding disease heterogeneity in AE and exploring microbiota-targeted interventions. However, direct AE-specific evidence remains limited, and many proposed mechanisms are currently supported primarily by observational studies, experimental models, or evidence extrapolated from related neuroimmune disorders. Future studies integrating longitudinal clinical cohorts, multi-omics profiling, and functional validation are needed to define key microbial functions, host immune responses, and their dynamic relationships in AE, thereby providing a stronger mechanistic foundation for future precision diagnostic and therapeutic strategies.

## 1 Introduction

AE comprises a heterogeneous group of immune-mediated inflammatory disorders of the central nervous system (Caballero-Ávila et al., 2025), characterized by autoantibodies targeting neuronal surface or synaptic antigens, aberrant immune-cell activation, and neurological dysfunction (Lu et al., 2025; Irani, 2024). In recent years, advances in neuroimmunology and autoantibody detection have substantially improved the diagnosis and treatment of AE (Sun et al., 2026). Nevertheless, current research has focused predominantly on autoantibody production, aberrant B-cell activation, and central neuroinflammatory processes, whereas the factors associated with disease susceptibility, interindividual heterogeneity, and recurrence remain incompletely understood (Afzali et al., 2024; Yu et al., 2023; Benamar et al., 2025).

The gut microbiota plays an important role in maintaining immune homeostasis, regulating host metabolism, and modulating neural function (Liu et al., 2026). Advances in gut–brain axis research have further expanded our understanding of how peripheral microbial signals may influence central nervous system function. In immune-mediated diseases, gut dysbiosis may involve not only alterations in microbial composition but also changes in microbial structural components, metabolic outputs, and secreted signals (Toydemir and Merey, 2026; Czaj et al., 2026). Microbe-associated molecules such as lipopolysaccharide (LPS) can activate inflammatory signaling through pattern recognition receptors and influence immune-cell activity (Gauthier et al., 2022). Microbial metabolites, including short-chain fatty acids (SCFAs), can modulate inflammatory responses by regulating T-cell differentiation, macrophage function, and immune tolerance (Lee et al., 2023). In addition, alterations in the gut microbiota may be accompanied by impaired intestinal barrier integrity, increasing host exposure to microbiota-derived signals and potentially affecting blood–brain barrier (BBB) function and central neuroinflammatory processes (Campagnoli et al., 2024).

However, research on the relationship between AE and the gut microbiota remains at an early stage. An integrated mechanistic framework linking microbiota-derived signals with peripheral immune activation, autoantibody generation, and central neuroinflammation is still lacking (Zhang et al., 2026; Dong et al., 2026). Increasing attention has also been directed toward the reciprocal influence of host immunity on the gut microbiota. The mucosal barrier, immunoglobulin A (IgA), antimicrobial peptides, and the local inflammatory milieu can influence microbial colonization, spatial organization, and ecological stability (Johnstone and Herzberg, 2022; Di Sabatino et al., 2023). Moreover, different phases of AE may be accompanied by distinct patterns of microbial alteration and immune remodeling (Piepgras et al., 2025). Examining AE from the perspective of bidirectional microbiota–neuroimmune crosstalk and dynamic disease evolution may therefore provide a useful framework for understanding disease heterogeneity while avoiding the assumption of a fixed or universally established pathogenic sequence.

Accordingly, this narrative review discusses current evidence regarding AE-associated alterations in the gut microbiota and neuroimmune dysregulation. Relevant literature was identified primarily through the Web of Science Core Collection and PubMed, with emphasis on studies published within the past 5 years and supplementation by selected landmark studies of mechanistic importance where appropriate. The evidence considered includes studies in patients with AE, AE-related animal models, patient-derived microbiota-transfer experiments, and mechanistic investigations in related neuroimmune disorders. Particular attention is given to distinguishing AE-specific evidence from findings extrapolated from multiple sclerosis, experimental autoimmune encephalomyelitis, and other neuroimmune conditions. We examine the potential mechanisms through which the gut microbiota may modulate neuroimmune responses and, conversely, how neuroimmune dysregulation may reshape the intestinal microbial environment.

We further discuss the potential dynamic relationships among gut dysbiosis, immune activation, and central neuroinflammation across different phases of AE and summarize emerging strategies targeting the gut microbiota–neuroimmune axis. Together, these considerations provide a conceptual framework for future mechanistic investigation and evaluation of microbiota-targeted approaches in AE. As this was a narrative review, no formal PRISMA-based systematic screening procedure or prespecified inclusion/exclusion criteria were applied; studies were selected according to their relevance to the scope of the review and their contribution to the mechanistic and translational discussion.

## 2 Gut microbial dysbiosis and neuroimmune homeostasis in AE

In recent years, advances in metagenomic sequencing and multi-omics technologies have drawn increasing attention to the potential role of the gut microbiota in neuroimmune disorders (Kumar et al., 2025; Yang et al., 2025). Research on AE-associated gut microbial alterations remains at an exploratory stage, with current evidence derived primarily from patient cohort studies, animal models, and microbiota-transfer experiments. Compared with healthy individuals, some patients with AE appear to exhibit alterations in gut microbial composition and functional profiles (Deng et al., 2025). However, the specific microbial changes reported across studies are not entirely consistent, potentially reflecting differences in age, dietary patterns, AE subtype, immunotherapy exposure, and disease phase. Accordingly, changes in individual bacterial taxa alone are unlikely to fully characterize AE-associated microbial alterations, and increasing attention has shifted toward ecological function and microbial metabolic capacity rather than purely taxonomic changes.

Several clinical studies have suggested that patients with AE may exhibit alterations in gut microbial diversity and community structure, accompanied by changes in short-chain fatty acid production, amino acid metabolism, and other microbial functional pathways (Wei et al., 2022; Shen et al., 2026; Ma et al., 2020). These findings suggest an association between alterations in the gut microbiota and the immune milieu of AE. However, whether these microbial changes participate in disease development, arise secondarily to immune dysregulation, or reflect context-dependent bidirectional interactions remains unclear.

Beyond alterations in microbial composition, impaired intestinal barrier function may represent an important interface between gut dysbiosis and host immune dysregulation. Dysbiosis may be accompanied by disruption of barrier integrity, thereby increasing host exposure to microbiota-derived signals and perturbing peripheral immune homeostasis (Wakabayashi et al., 2026; Chen et al., 2025; Oami et al., 2025; Acioglu and Elkabes, 2025). In models of other inflammatory and autoimmune diseases, microbial signals have been shown to modulate the functional states of macrophages, dendritic cells, and T cells and to influence inflammatory cytokine production and immune tolerance (Marrocco and Ortiz, 2022; Wolff et al., 2023), while peripheral inflammatory states may also compromise BBB integrity (Beltran-Velasco and Clemente-Suárez, 2025). Direct evidence for these mechanisms in AE, however, remains limited, and their disease-specific relevance requires further validation.

Immune dysregulation in AE involves multiple abnormalities, including alterations in the T helper 17/regulatory T-cell (Th17/Treg) balance, aberrant B-cell activation, enhanced plasma-cell differentiation, and autoantibody production (Aso et al., 2023; Sivalingam, 2025; Räuber et al., 2025). In models of other autoimmune and neuroimmune disorders, selected microbial metabolites have been shown to modulate immune tolerance, whereas dysbiosis-associated changes in the metabolic environment may influence the maintenance of immune homeostasis (Kim, 2023; Lv et al., 2025). These findings provide mechanistic context for considering microbiota–immune interactions in AE, but whether the same mechanisms operate directly in AE remains to be established. Conversely, host immunity can influence microbial composition and spatial organization through IgA, antimicrobial peptides, and inflammatory mediators (León and Francino, 2022), supporting the possibility of bidirectional interactions between the gut microbiota and the immune system.

Importantly, current evidence linking AE to alterations in the gut microbiota is derived largely from cross-sectional observations, animal models, and exploratory mechanistic studies. Existing data are therefore insufficient to determine whether microbial alterations contribute to AE pathophysiology, represent secondary consequences of immune dysfunction, or arise as part of a reciprocal feedback process. Moreover, different disease phases may be accompanied by distinct degrees of microbial remodeling and immune alteration, making single-time-point analyses poorly suited to capturing the dynamic nature of these interactions.

Potential confounding factors inherent to human microbiome research should also be carefully considered when interpreting AE-associated microbial signatures. Host-related variables, including age, sex, body mass index (BMI), dietary patterns, and geographic environment, can substantially influence gut microbial composition and metabolic function. Differences in these factors across study cohorts may therefore account for some of the microbial features attributed to AE (Nearing et al., 2021; Johnson et al., 2019). Disease-related factors, including disease severity, hospitalization, constipation, and other gastrointestinal symptoms, as well as treatment exposures such as antibiotics, glucocorticoids, immunotherapy, antiseizure medications, and proton pump inhibitors, may also independently alter the gut microbiota and complicate interpretation of AE-associated microbial changes (Nel Van Zyl et al., 2022; Puértolas-Balint et al., 2025).

Technical variation may further contribute to inconsistencies across studies. Differences in sample collection and storage, DNA extraction procedures, sequencing platforms, and bioinformatic pipelines can influence microbiome profiles and limit direct comparability across datasets (Tett et al., 2021). Future studies should therefore incorporate longitudinal cohorts, multi-omics profiling, and functional validation while rigorously controlling for relevant clinical and technical variables. Such approaches will be important for clarifying the relationships among gut microbial alterations, clinical phenotypes, immune dysregulation, and neuroinflammatory processes in AE.

## 3 Molecular mechanisms underlying gut microbiota–neuroimmune crosstalk

The relationship between the gut microbiota and the host immune system is bidirectional. Gut microorganisms can modulate immune-cell function through structural components, metabolites, and secreted signals, with potential effects on blood–brain barrier integrity and central neuroinflammation. Conversely, the neuroimmune system may reshape microbial composition and ecological stability through neuroendocrine–immune regulatory networks and changes in the inflammatory milieu. In the context of AE, such crosstalk may provide a potential mechanistic link among intestinal dysbiosis, peripheral immune activation, and neuroinflammation and may be relevant to AE pathophysiology.

### 3.1 Mechanisms by which the gut microbiota regulates neuroimmune responses

The gut microbiota can influence host neuroimmune regulation through multiple classes of microbial signals, including structural components, metabolites, secreted factors, and nucleic acid-derived molecules. These signals can act on distinct immune-cell populations and signaling pathways, thereby modulating inflammatory responses, immune tolerance, and BBB function and potentially contributing to AE-associated neuroimmune dysregulation.

Current evidence can broadly be classified into three levels. The first comprises AE-specific human studies, including analyses of microbial composition, metabolic profiles, and their associations with clinical phenotypes. The second includes AE-related experimental evidence, such as transplantation of patient-derived microbiota and validation in AE-related animal models. The third consists of indirect mechanistic evidence derived from multiple sclerosis (MS), experimental autoimmune encephalomyelitis (EAE), and other neuroimmune disease models. The second category provides disease-relevant experimental support, whereas findings in the third category require cautious extrapolation to AE. To provide an integrated overview of microbiota-derived signals involved in neuroimmune regulation, the major pathways are illustrated in Figure 1, while the principal signal classes and their functional characteristics are summarized in Table 1 (Wei et al., 2025; Erickson et al., 2023; Cai et al., 2021; Kim et al., 2023; Kemter et al., 2023; Pearson et al., 2024; Bersch et al., 2021; Ferrand et al., 2019; Zou et al., 2021; Hu et al., 2022; Duscha et al., 2020; Wang G. et al., 2024; Hezaveh et al., 2022; Rothhammer et al., 2018; Paik et al., 2022; Li et al., 2021; Campbell et al., 2020; Chiaranunt et al., 2023; Karmakar et al., 2016; Mottawea et al., 2025; Ha et al., 2020; Han et al., 2019; Gross et al., 2024; Luo et al., 2021; Xu et al., 2022; Nunes et al., 2024; Ugolini et al., 2018; Campos et al., 2017).

**Figure 1:**
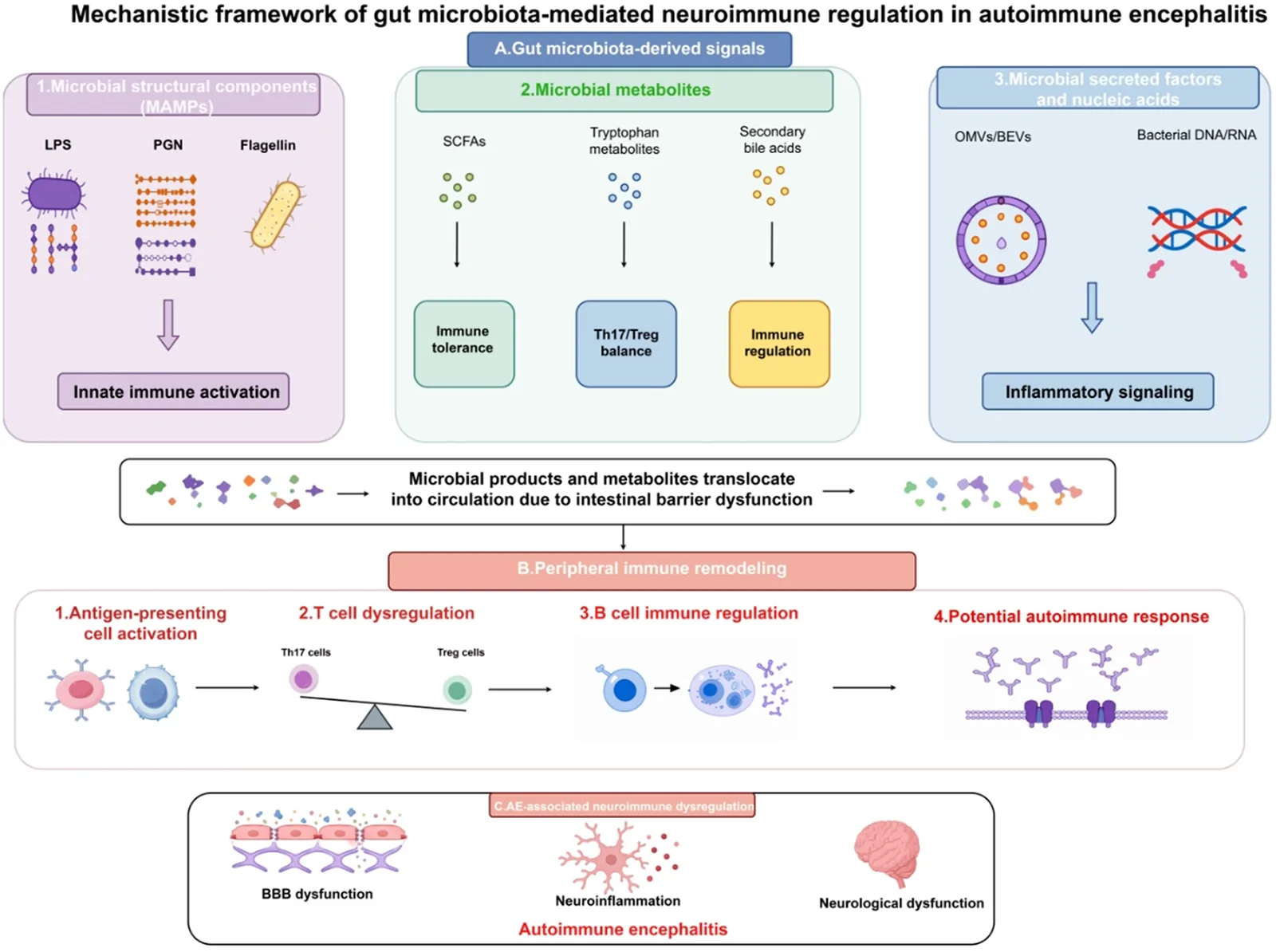
Mechanistic framework of gut microbiota-mediated neuroimmune regulation in autoimmune encephalitis. **(A)** Gut microbiota-derived signals, including microbial structural components (MAMPs), microbial metabolites, and microbial secreted factors and nucleic acids, regulate innate immune activation, immune tolerance, Th17/Treg balance, and inflammatory signaling. Intestinal barrier dysfunction may facilitate the translocation of microbial products and metabolites into the circulation. **(B)** Peripheral immune remodeling involves antigen-presenting cell activation, T-cell dysregulation, B-cell immune regulation, and the subsequent development of potential autoimmune responses. **(C)** Autoimmune encephalitis-associated neuroimmune dysregulation is characterized by blood-brain barrier dysfunction, neuroinflammation, and neurological dysfunction.

**Table 1:** Major gut microbiota-derived signals involved in neuroimmune regulation and their potential relevance to AE.

Signal category	Representative molecule/factor	Primary microbial source	Major targets/signaling pathways	Principal immunoregulatory functions	Potential relevance to AE	Evidence level/source	References
Microbial structural components	LPS	Outer membrane of Gram-negative bacteria	TLR4/MyD88/NF-κB; macrophages, dendritic cells, and cerebrovascular endothelial cells	Induces TNF-α, IL-1β, IL-6 and other inflammatory mediators, promoting innate immune activation and endothelial inflammation	Under conditions of barrier disruption, gut-derived LPS may promote systemic inflammation, microglial activation, and BBB dysfunction, thereby creating a pro-inflammatory milieu relevant to AE-associated neuroinflammation	Indirect evidence: gut–brain axis animal studies and BBB mechanistic studies; no direct evidence in AE	Nearing et al., 2021; Johnson et al., 2019
Microbial structural components	LTA	Cell wall of Gram-positive bacteria	TLR2/MyD88, PI3K/AKT, NF-κB/MAPK; epithelial cells, macrophages, and dendritic cells	Depending on bacterial species and molecular structure, may induce inflammatory cytokine production or restrain excessive dendritic cell activation	May contribute to peripheral immune activation or immune tolerance following microbial dysbiosis; however, its role in AE remains unclear	Indirect evidence: cell-based mechanistic studies and human immune cell studies; no direct evidence in AE	Nel Van Zyl et al., 2022; Puértolas-Balint et al., 2025
Microbial structural components	Flagellin	Flagellated commensal and pathogenic bacteria	TLR5/MyD88; CD11c^+^ antigen-presenting cells, dendritic cells, ILC3s, and T cells	Regulates IL-23–IL-22 axis, mucosal barrier integrity, and dendritic cell function; effects are context dependent	May indirectly influence AE-associated peripheral immune environment by modulating intestinal barrier integrity, antigen presentation, and autoreactive T-cell activation threshold	Indirect evidence: animal mechanistic studies and autoimmune disease models; no direct evidence in AE	Tett et al., 2021; Wei et al., 2025
Microbial structural components	Peptidoglycan (PGN) and PGN-derived fragments	Cell walls of Gram-positive and Gram-negative bacteria	NOD1/NOD2–RIP2–NF-κB/MAPK; epithelial cells and monocytes/macrophages	Different PGN fragments induce distinct inflammatory programs; NOD2 also contributes to barrier homeostasis and immune tolerance toward commensal microbes	May influence peripheral immune homeostasis through intestinal barrier regulation, myeloid activation, and host–microbiota recognition; evidence in AE is lacking	Indirect evidence: macrophage mechanistic studies and gene-modified animal studies; no direct evidence in AE	Erickson et al., 2023; Cai et al., 2021
Microbial metabolites	SCFAs	Produced by anaerobic bacterial fermentation of dietary fiber	GPR43/GPR109A, HDAC inhibition, and p38 MAPK; Tregs, regulatory B cells, and macrophages	Promotes Treg differentiation and IL-10-mediated immunoregulation, enhances immune tolerance, and suppresses excessive inflammatory responses	Altered SCFA production may influence peripheral immune tolerance and barrier stability relevant to AE-associated autoimmunity, although current evidence is mainly derived from other autoimmune diseases	Indirect evidence: MS clinical/mechanistic studies and autoimmune disease models; no direct evidence in AE	Kim et al., 2023; Kemter et al., 2023; Pearson et al., 2024
Microbial metabolites	Tryptophan metabolites	Lactobacillus, Clostridium, and other tryptophan-metabolizing gut bacteria	AhR; epithelial cells, ILC3s, T cells, macrophages, microglia, and astrocytes	Promotes IL-22 production and barrier protection, regulates Th17/Treg balance, and reshapes myeloid cell function in a ligand- and cell-dependent manner	May influence AE-associated neuroinflammation by regulating intestinal barrier function, peripheral immune tolerance, and microglia–astrocyte interactions	Indirect evidence:neuroinflammation and autoimmune disease models; no direct evidence in AE	Bersch et al., 2021; Ferrand et al., 2019; Zou et al., 2021
Microbial metabolites	Secondary bile acids and derivatives	Microbial transformation of primary bile acids through deconjugation, dehydroxylation, and epimerization	RORγt, NR4A1, FXR, TGR5, and VDR-related pathways	Metabolite-specific inhibition of Th17 differentiation, promotion of peripheral Treg generation, or modulation of Treg function	Altered bile acid metabolism may disrupt Th17/Treg balance and peripheral immune tolerance, thereby indirectly influencing AE-associated autoimmunity	Indirect evidence: microbial metabolism studies, immune cell assays, and colonization animal studies; no direct evidence in AE	Hu et al., 2022; Duscha et al., 2020; Wang G. et al., 2024
Microbial metabolites	Microbiota-derived extracellular ATP and other purinergic signals	Multiple intestinal bacteria; ATP in inflamed tissues may also originate from damaged host cells	P2X7R–NLRP3–caspase-1; macrophages, neutrophils, and glial cells	Promotes inflammasome assembly, IL-1β maturation and release, and regulates inflammatory homeostasis	Microbial ATP may promote peripheral immune activation; however, evidence that microbiota-derived ATP directly enters CNS and drives AE is lacking	Indirect evidence: gut microbiota–immune animal studies and cell-based mechanistic studies; no direct evidence in AE	Hezaveh et al., 2022; Rothhammer et al., 2018
Microbial secreted factors	OMVs/BEVs	Primarily Gram-negative bacteria; some Gram-positive bacteria also release extracellular vesicles	TLR2/TLR4–NF-κB/MAPK; endothelial cells, macrophages, brain monocytes, and microglia	Deliver LPS, lipoproteins, proteins, DNA and RNA, mediating trans-barrier and long-distance immune signaling	May serve as carriers linking gut microbial dysbiosis with systemic immune activation and CNS inflammation; their role in AE remains to be established	Indirect evidence: barrier models, animal tracing studies, and neuroinflammation models; no direct evidence in AE	Paik et al., 2022; Li et al., 2021; Campbell et al., 2020
Microbial nucleic acid signals	Bacterial DNA and DNA-containing bacterial extracellular vesicles	Bacterial lysis, active secretion, or extracellular vesicle-mediated release	Endosomal TLR9–MyD88 and cytosolic cGAS–STING–TBK1–IRF3 pathways	Induces inflammatory cytokines and type I interferon responses	May enhance systemic innate immunity and potentially amplify AE-associated autoimmunity after barrier disruption	Indirect evidence: macrophage studies, gene-modified animal studies, and inflammation models; no direct evidence in AE	Chiaranunt et al., 2023; Karmakar et al., 2016; Mottawea et al., 2025
Microbial nucleic acid signals	Bacterial RNA and extracellular vesicle-associated RNA	Live bacteria, bacterial lysis products, and bacterial extracellular vesicles	TLR8; TLR7 and TLR3 in specific contexts	Induces IL-12, IL-6, TNF-α and type I interferon responses; influences Tfh differentiation and humoral immunity	May contribute to abnormal humoral immune environments associated with AE; currently remains a mechanistic hypothesis	Indirect evidence: human immune cell studies and animal models; no direct evidence in AE	Ha et al., 2020; Han et al., 2019; Gross et al., 2024

Microbial structural components represent an important class of signals through which the host senses changes in the intestinal microbial environment. Microbe-associated molecular patterns (MAMPs), including lipopolysaccharide (LPS), lipoteichoic acid (LTA), flagellin, and peptidoglycan (PGN), can be recognized by host pattern recognition receptors (PRRs) and induce immune-cell activation. LPS primarily activates the TLR4/MyD88/NF-κB signaling axis, promoting the production of inflammatory mediators such as TNF-α, IL-1β, and IL-6 by macrophages and dendritic cells. LTA, flagellin, and PGN participate in innate immune regulation through TLR2, TLR5, and NOD-like receptor pathways, respectively. Under physiological conditions, these microbial signals contribute to host immune homeostasis. In the setting of dysbiosis and impaired intestinal barrier integrity, however, increased exposure to microbiota-derived molecules may enhance peripheral inflammatory signaling, promote sustained immune-cell activation, and potentially affect blood–brain barrier integrity after entering the systemic circulation. Microbial structural components may therefore represent potential upstream signals linking intestinal dysbiosis to peripheral and neuroimmune responses, although their specific contribution to AE remains to be established.

The gut microbiota can also modulate immune responses through its metabolic products. Short-chain fatty acids (SCFAs) are major products of microbial fermentation of dietary fiber. Among them, butyrate and propionate can regulate immune-cell function through G-protein-coupled receptors (GPCRs) and inhibition of histone deacetylases (HDACs), thereby promoting regulatory T-cell (Treg) differentiation and immune tolerance. Microbial tryptophan metabolites can act as ligands for the aryl hydrocarbon receptor (AhR), influencing the Th17/Treg balance and IL-22-associated barrier-protective responses. Microbially modified bile acids can further regulate T-cell and macrophage function through pathways involving FXR and TGR5. Other microbiota-associated metabolic signals, including ATP and lactate, may also participate in inflammatory regulation. Microbial metabolites may reshape immune-cell functional states and thereby influence immune susceptibility and homeostasis. However, much of the mechanistic evidence supporting these pathways is derived from MS, EAE, and other immune-mediated disease models, and their relevance to AE requires further validation.

Microbial secreted factors and nucleic acid-derived molecules may additionally function as long-range immunoregulatory signals. Outer membrane vesicles (OMVs) can carry microbial components, including LPS, proteins, and nucleic acids, and facilitate their delivery to host cells, thereby modulating TLR-related inflammatory signaling. Bacterial DNA and RNA can also activate type I interferon responses and inflammatory pathways through TLR9, TLR3/TLR7, and cGAS–STING signaling. Although direct evidence for these mechanisms in AE remains limited, studies from broader microbiome and inflammatory research suggest that microbiota-derived secreted factors may extend microbial signaling beyond the local intestinal environment and contribute to systemic immune regulation.

### 3.2 Reciprocal regulation of the gut microbiota by the neuroimmune system

In parallel with the influence of the gut microbiota on neuroimmune states, the neuroimmune system may reciprocally shape the intestinal microbial environment through neuroendocrine–immune networks. Neuroimmune regulation extends beyond microglial activation and inflammatory responses within the central nervous system and encompasses systemic processes involving neuronal signaling, peripheral immune mediators, and neuroendocrine regulation (Kaur et al., 2025; Biswas, 2023). In AE-associated states, autoantibody-mediated immune dysregulation, glial activation, and persistent inflammation may be accompanied by alterations in intestinal barrier function and the local microbial environment, raising the possibility that at least some microbiota changes represent secondary consequences of neuroimmune dysfunction (Wu et al., 2024).

Neuroendocrine regulation represents an important pathway linking neuroimmune activity to intestinal ecology. The autonomic nervous system, particularly vagal and sympathetic signaling, can influence the microbial colonization environment by modulating epithelial barrier function and local immune responses (Macpherson et al., 2023; Chen and Tang, 2026). The vagus nerve-mediated cholinergic anti-inflammatory pathway regulates macrophage inflammatory responses through the α7 nicotinic acetylcholine receptor (α7nAChR), suggesting that neural signals may indirectly influence microbial homeostasis through immune modulation (Keever et al., 2024). In parallel, alterations in glucocorticoid signaling driven by the hypothalamic–pituitary–adrenal (HPA) axis can affect intestinal epithelial permeability, immune function, and the local metabolic environment, thereby influencing gut microbial composition (Bertollo et al., 2025; Rusch et al., 2023). Dysregulation of these pathways may be associated with intestinal microbial alterations in AE, although their disease-specific roles remain to be established.

Neuroinflammation-associated immune mediators and disturbances in redox homeostasis may also participate in microbial ecosystem remodeling. During AE, central and peripheral immune activation can involve microglial activation, aberrant T-cell responses, B-cell-mediated autoantibody production, and increased release of inflammatory cytokines, including IL-6, TNF-α, IL-1β, and IL-17 (León and Francino, 2022; Touil et al., 2023). A persistent inflammatory milieu may destabilize epithelial tight junctions, increase the risk of intestinal barrier dysfunction, and alter microbial ecological niches. In parallel, inflammation-associated changes in redox status may affect the intestinal redox environment, epithelial integrity, and microbial competition through altered production of reactive oxygen species (ROS), reactive nitrogen species (RNS), and nitric oxide (NO), potentially favoring the expansion of selected microbial populations while reducing certain commensal taxa (Herrera-Quintana et al., 2026; Steinert et al., 2025; Shandilya et al., 2021).

Oxidative stress may further modulate immune-cell activation and inflammatory responses through mitochondrial dysfunction and redox-sensitive pathways, including NF-κB, the NLRP3 inflammasome, and Nrf2, thereby influencing interactions between microbiota-derived signals and host immunity (Saha et al., 2022; Tastan et al., 2022). Dysregulated signaling mediated by ROS, RNS, and NO has been implicated in immune regulation and tissue injury across a range of neuroinflammatory disorders, providing additional mechanistic context for the potential links among the microbiota, immune responses, and neuroinflammation (Naderian et al., 2026). Importantly, direct evidence for these redox-related mechanisms in AE remains limited. Current concepts are derived largely from other inflammatory diseases, MS/EAE, and broader neuroinflammatory models; therefore, their specific contribution to microbiota–neuroimmune crosstalk in AE requires further validation.

The BBB and intestinal barrier may together constitute a bidirectional interface linking the peripheral microbial environment with central neuroimmune responses. Existing studies indicate that gut dysbiosis can influence peripheral immune states through microbial structural components and metabolites and may be associated with BBB dysfunction and neuroinflammation (You et al., 2025; Szukiewicz et al., 2026). Conversely, persistent neuroimmune activation may influence intestinal barrier integrity through inflammatory mediator release, neuroendocrine dysregulation, and peripheral immune remodeling, thereby altering the ecological conditions that shape microbial colonization (Mosaddeghi-Heris et al., 2026; Salvo-Romero et al., 2022).

Collectively, the potential reciprocal regulation of the gut microbiota by the neuroimmune system further highlights the bidirectional nature of the gut–brain axis. Microbial abnormalities observed in AE may be shaped by multiple factors, including central neuroinflammation, immune-mediated injury, and neuroendocrine disturbances, and may interact dynamically with disease-associated immune dysregulation. Nevertheless, direct evidence demonstrating neuroimmune-driven remodeling of the gut microbiota in AE remains scarce, and the proposed feedback network should currently be regarded as a mechanistic framework rather than an established AE-specific pathway. Future longitudinal clinical studies integrating multi-omics profiling and functional validation will be necessary to clarify how neuroimmune–microbiota interactions vary across different phases of AE.

## 4 Dynamic evolution of gut microbiota–neuroimmune crosstalk during AE

Based on the heterogeneous evidence currently available from studies of patients with AE, animal models, and related neuroimmune disorders, we propose a phase-based conceptual model to facilitate understanding of gut microbiota–neuroimmune crosstalk during AE. This model postulates that AE-associated microbial alterations, peripheral immune activation, and central neuroinflammation may be dynamically interconnected across three broadly defined phases: disease initiation, progression, and the chronic phase. Importantly, this framework should not be interpreted as an established linear pathophysiological sequence. Rather, it represents a working hypothesis derived from current evidence and is intended to integrate biological events and potential feedback mechanisms that may occur at different points during the disease course (Figure 2).

**Figure 2:**
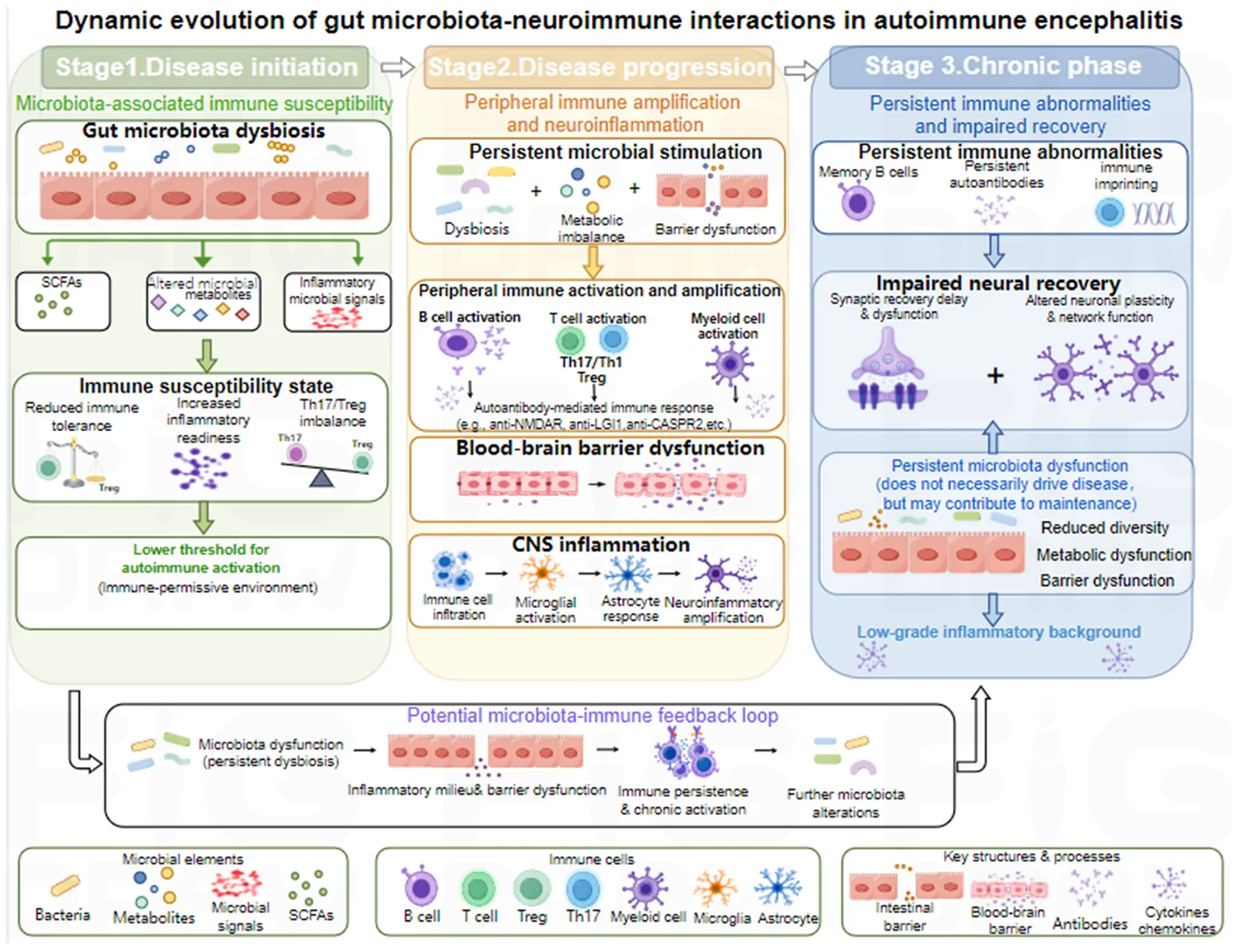
Hypothetical phase-based model of the dynamic evolution of gut microbiota–neuroimmune interactions in autoimmune encephalitis. This figure illustrates a proposed working conceptual model of the dynamic evolution of gut microbiota-neuroimmune interactions during autoimmune encephalitis (AE). Based on currently available heterogeneous evidence from studies in patients with AE, experimental models, and related neuroimmune disorders, the disease course is conceptually divided into three interconnected phases: disease initiation, disease progression, and the chronic phase. During disease initiation, gut microbiota dysbiosis and microbial metabolic alterations may be associated with an immune-susceptible state by influencing barrier integrity, immune tolerance, and inflammatory tone. During disease progression, persistent microbial signals, peripheral immune amplification, blood-brain barrier (BBB) dysfunction, and central nervous system (CNS) inflammation may interact to promote neuroinflammatory responses and autoimmune injury. During the chronic phase, persistent immune abnormalities, impaired neural recovery, and sustained microbiota dysfunction may be associated with disease persistence and neurological sequelae. A potential microbiota-immune feedback loop is included to indicate that chronic inflammation and microbiota alterations may mutually influence one another over time. This figure represents a hypothesis-generating conceptual framework rather than an established linear pathogenic cascade, and the exact temporal sequence and causal relationships remain to be validated. SCFAs, short-chain fatty acids; BBB, blood-brain barrier; CNS, central nervous system.

### 4.1 Disease initiation: dysbiosis and the establishment of an immune-susceptible state

Within the proposed framework, alterations in the gut microbiota may be associated with changes in peripheral immune regulation that increase susceptibility to autoimmune activation. Current evidence suggests that gut dysbiosis may influence host immune homeostasis by altering microbial community structure, increasing exposure to proinflammatory microbial signals, and modifying immunoregulatory networks, potentially creating an immune environment more permissive to autoimmunity.

Gut microbiota studies in AE have thus far focused predominantly on anti-N-methyl-D-aspartate receptor encephalitis (anti-NMDAR encephalitis). Gong et al. (2019) were among the first to report alterations in the gut microbiota of patients with anti-NMDAR encephalitis, identifying reduced microbial diversity and altered community composition and suggesting that the disease state may be accompanied by intestinal microbial remodeling. In contrast, Herken et al. (2019) did not detect marked overall differences in gut microbial composition between patients and healthy controls in an independent cohort, indicating that AE-associated microbial signatures may be influenced by disease phase, treatment status, diet, sampling-related factors, and other sources of interindividual variation.

Patient-derived microbiota transplantation has provided experimental support for a potential role of the gut microbiota in AE-related immune regulation. Chen et al. (2020) reported that microbiota derived from patients with anti-NMDAR encephalitis increased the proportion of Th17 cells in recipient mice and was accompanied by behavioral abnormalities, suggesting that disease-associated microbial communities may influence peripheral immune balance and neurobehavioral phenotypes. Subsequent studies identified microbial and metabolic abnormalities in patients with anti-NMDAR encephalitis before treatment, with selected microbial features correlating with disease severity and clinical outcomes (Gong et al., 2021). Additional animal experiments showed that patient-derived microbiota reduced SCFA levels, increased inflammatory cytokine production, decreased the expression of BBB tight-junction proteins, and exacerbated neuroinflammation and behavioral abnormalities in a GluN1 active-immunization model (Gong et al., 2024). Together, these findings suggest that microbiota alterations may modify host susceptibility to autoimmune challenge by influencing immune homeostasis and barrier function. Whether such alterations directly participate in AE initiation, however, remains to be established.

Beyond AE-specific evidence, studies in MS, EAE, and other autoimmune models further indicate that alterations in microbial composition and metabolic function can influence T-cell immune balance and susceptibility to neuroinflammation (Zhou et al., 2022; Cekanaviciute et al., 2017; Han et al., 2024; Lin et al., 2024; Hang et al., 2019). Nevertheless, these mechanisms have largely been defined in related disease models, and their applicability to the initiation phase of AE requires disease-specific validation.

### 4.2 Disease progression: peripheral immune amplification and neuroinflammation

Within the proposed model, persistent microbial dysregulation may continue to influence the peripheral immune milieu and become associated with amplification of autoimmune responses and central neuroinflammation. In patients with anti-NMDAR encephalitis, alterations in the gut microbiota and microbial metabolism have been associated with disease severity, whereas animal studies indicate that patient-derived microbiota can enhance inflammatory responses and impair BBB stability (Gong et al., 2021, 2024). These findings suggest that gut dysbiosis may contribute to shaping the immune environment during disease progression, although its precise role remains uncertain.

Peripheral immune amplification is an important feature of clinically active AE. Single-cell immunoprofiling and cerebrospinal fluid studies have demonstrated increased populations of antibody-secreting cells and class-switched memory B cells, together with enhanced inflammatory signaling, during the acute phase of anti-NMDAR encephalitis, indicating coordinated involvement of peripheral and central immune responses (Jiang et al., 2022; Li et al., 2024). In addition, germinal center structures within peripheral lymphoid tissues and ovarian teratomas can support the maturation of NMDAR-reactive B cells and production of NR1-IgG, suggesting that peripheral immune compartments may contribute to sustained autoantibody generation (Al-Diwani et al., 2022).

Within this process, gut dysbiosis may modulate the inflammatory context by influencing immune-cell activation states. However, there is currently no evidence demonstrating that specific microbial communities directly determine the generation of particular autoantibody-producing clones. The microbiota should therefore be regarded primarily as a potential modifier of the immunological environment rather than a direct determinant of the autoimmune effector response.

Persistent peripheral immune activation may be accompanied by disruption of BBB integrity, potentially facilitating the access of immune mediators and cells to the central nervous system. Using peripheral blood mononuclear cells from patients with anti-NMDAR encephalitis, Shu et al. (2023) established a humanized model in which patient-derived immune cells induced BBB disruption and an encephalitis-like phenotype. Clinical studies have further shown that impaired BBB integrity is associated with disease severity, intrathecal IgG synthesis, and clinical outcome (Yu et al., 2021). Considered together with BBB alterations observed in microbiota-transfer models, these findings raise the possibility that microbial dysregulation may influence BBB vulnerability, although the underlying mechanisms remain to be clarified.

Within the central nervous system, infiltrating peripheral immune cells, autoantibodies, and resident innate immune responses may collectively contribute to a neuroinflammatory environment. Chang et al. (2023) reported elevated cerebrospinal fluid levels of soluble triggering receptor expressed on myeloid cells 2 (sTREM2) in patients with anti-NMDAR encephalitis, with higher levels associated with BBB disruption and greater disease severity, supporting the involvement of microglial activation. Neuropathological studies have additionally demonstrated lymphocytic infiltration and microglial activation in brain tissue, indicating that peripheral immune input and central innate immune responses may act together in neuroinflammation (Zrzavy et al., 2021).

Autoantibody-mediated synaptic dysfunction represents an important downstream effector mechanism in anti-NMDAR encephalitis. Structural and mechanistic studies have shown that patient-derived autoantibodies bind to the GluN1 subunit, promote clustering and internalization of NMDARs, and thereby reduce receptor density and function at the neuronal surface (Wang H. et al., 2024). Within the present conceptual framework, the progression phase may therefore involve dynamic interactions among microbial dysregulation, peripheral immune amplification, BBB dysfunction, central neuroinflammation, and synaptic dysfunction rather than a fixed linear cascade.

### 4.3 Chronic phase: aberrant immune memory and microbial functional dysregulation

Within the proposed chronic phase, aberrant immune memory, persistent antibody-related immune activity, and incomplete neurological recovery may coexist. Importantly, direct evidence supporting a role for the gut microbiota in AE chronicity remains limited. Microbial dysregulation should therefore currently be considered a potential contributor to a persistent proinflammatory background rather than an established driver of chronic disease.

Although some patients improve clinically after treatment of the acute phase, underlying immune abnormalities may not completely resolve. Studies have shown that subsets of patients retain antibody-secreting cells, memory B-cell populations, and expanded B-cell receptor (BCR) clonotypes after treatment, suggesting that persistent immune activity in peripheral and central immune compartments may be associated with prolonged disease courses (Jiang et al., 2022; Li et al., 2024; Al-Diwani et al., 2022; Feng et al., 2020; Theorell et al., 2024).

Delayed recovery from antibody-mediated neuronal dysfunction may represent another feature of persistent neurological impairment. Patient-derived antibodies can induce seizure phenotypes in animal models, while impairments in learning and memory and abnormalities in neurogenesis may persist even after antibody exposure has ceased, suggesting that synaptic recovery and neural-network remodeling may lag behind the resolution of inflammation (Taraschenko et al., 2021, 2024). Together with the ability of NMDAR antibodies to promote receptor clustering and internalization, these findings suggest that persistent neurological symptoms may partly reflect delayed restoration of neuronal and synaptic function (Wang H. et al., 2024).

Compared with the evidence supporting persistent immune and neuronal abnormalities, evidence for a role of the gut microbiota during chronic or persistent AE remains relatively weak. Previous studies have associated lower microbial diversity with poorer clinical outcomes, while transplantation of patient-derived microbiota has been shown experimentally to reduce SCFA levels, enhance inflammatory responses, and impair BBB stability. These observations raise the possibility that persistent microbial functional dysregulation may be associated with a low-grade inflammatory milieu and impaired immune recovery and may interact with residual immune abnormalities. Whether the gut microbiota directly contributes to AE chronicity or recurrence remains unknown and will require validation in long-term longitudinal cohorts and microbiota-targeted intervention studies.

## 5 Gut microbiota features and evidence heterogeneity across AE subtypes

AE is not a single disease entity but rather a heterogeneous group of disorders associated with autoantibodies targeting distinct neuronal antigens and characterized by diverse clinical manifestations and immunopathological mechanisms. Well-recognized AE subtypes include anti-NMDAR encephalitis, LGI1-antibody encephalitis, CASPR2-antibody encephalitis, GABA-B receptor-antibody encephalitis, AMPAR-antibody encephalitis, and seronegative AE. These subtypes differ substantially in patient age, clinical phenotype, antibody characteristics, underlying immune mechanisms, and disease context (Bhargava et al., 2020). It therefore remains unclear whether gut microbiota–neuroimmune crosstalk reflects shared biological processes across AE subtypes or whether different subtypes are associated with distinct microbial signatures.

Current research on AE-associated gut microbiota alterations has focused predominantly on anti-NMDAR encephalitis. Existing studies have identified changes in gut microbial composition and function in patients with anti-NMDAR encephalitis, in some cases accompanied by alterations in microbial metabolism and immune phenotypes (Naderian et al., 2026; Gong et al., 2021). In addition, transplantation experiments using patient-derived microbiota suggest that disease-associated microbial communities may influence the Th17/Treg balance, the inflammatory milieu, and blood–brain barrier function (Mosaddeghi-Heris et al., 2026; Gong et al., 2024). However, anti-NMDAR encephalitis is characterized primarily by aberrant B-cell activation, autoantibody production, and antibody-mediated synaptic dysfunction. Whether microbiota-related mechanisms identified in this subtype are applicable to other forms of AE therefore requires cautious interpretation.

By comparison, gut microbiota research in LGI1- and CASPR2-antibody encephalitis remains limited. Available evidence suggests that patients with LGI1-antibody encephalitis may exhibit alterations in gut microbial composition and function, together with abnormalities in selected microbial metabolic pathways (Ma et al., 2020). Moreover, LGI1- and CASPR2-antibody encephalitis differ from anti-NMDAR encephalitis in age distribution, mechanisms of antibody-mediated pathology, and patterns of immune-cell involvement, raising the possibility that microbiota–neuroimmune interactions may exhibit subtype-specific features (Xu et al., 2023). At present, however, there is insufficient evidence to establish that microbiota alterations associated with LGI1- or CASPR2-antibody encephalitis play a causal role in disease development. Such changes may instead reflect the disease state, underlying immune dysregulation, treatment exposure, or a combination of these factors.

For GABA-B receptor-antibody encephalitis, AMPAR-antibody encephalitis, and seronegative AE, direct studies specifically characterizing the gut microbiota are currently lacking. These subtypes differ considerably in clinical presentation, tumor association, and immunological background. For example, GABA-B receptor- and AMPAR-antibody encephalitis are more frequently associated with underlying malignancy, whereas seronegative AE exhibits greater disease heterogeneity because of the absence of a defined antibody biomarker (Li et al., 2022; Wang et al., 2021). Consequently, gut microbial alterations in these subtypes may be influenced by multiple factors, including age, tumor status, immunotherapy exposure, and disease phase, and should not be inferred directly from findings obtained in anti-NMDAR or LGI1-antibody encephalitis.

Overall, gut microbiota–neuroimmune crosstalk may involve several biological processes that are potentially shared across AE subtypes, including alterations in barrier function, immunomodulation by microbial metabolites, and remodeling of the inflammatory microenvironment. Nevertheless, differences in target antigens, patterns of immune-cell involvement, and disease context may also give rise to subtype-specific microbial features. Because current gut microbiota research in AE remains concentrated in only a limited number of antibody-defined subtypes, future studies integrating antibody stratification with microbiome profiling, metabolomics, and immune phenotyping will be essential for distinguishing potentially shared mechanisms from subtype-specific microbial signatures and for identifying clinically meaningful microbiome biomarkers across the AE spectrum (Xu et al., 2024).

## 6 Therapeutic strategies targeting the gut microbiota and their translational potential

As gut microbiota–neuroimmune crosstalk has gained increasing attention, strategies targeting microbial dysregulation have emerged as a potential area of investigation in AE. Unlike conventional immunotherapies, which primarily target autoantibody production, B-cell activation, and inflammatory signaling, microbiota-directed approaches aim more broadly to modulate disease-associated microbial structure, metabolic function, and host–microbe interactions. Such interventions may influence peripheral immune activity, barrier function, and the neuroinflammatory milieu. However, microbiota-targeted interventions in AE remain largely at the mechanistic, experimental, or preclinical stages, and their clinical efficacy in AE has not been established. The overall therapeutic framework is illustrated in Figure 3, while the principal mechanisms, evidence base, and limitations of individual strategies are summarized in Table 2 (Han et al., 2024; Lin et al., 2024; Wastyk et al., 2021; Alexander et al., 2024; Ozgun Acar, 2025; Shi et al., 2024; Montgomery et al., 2024; Kang et al., 2024; Guimaraes et al., 2023; Federici et al., 2022; Duan et al., 2019; Ferreira et al., 2024; Lancaster, 2022; Abboud et al., 2021; Graus et al., 2021; Sinha et al., 2015).

**Figure 3:**
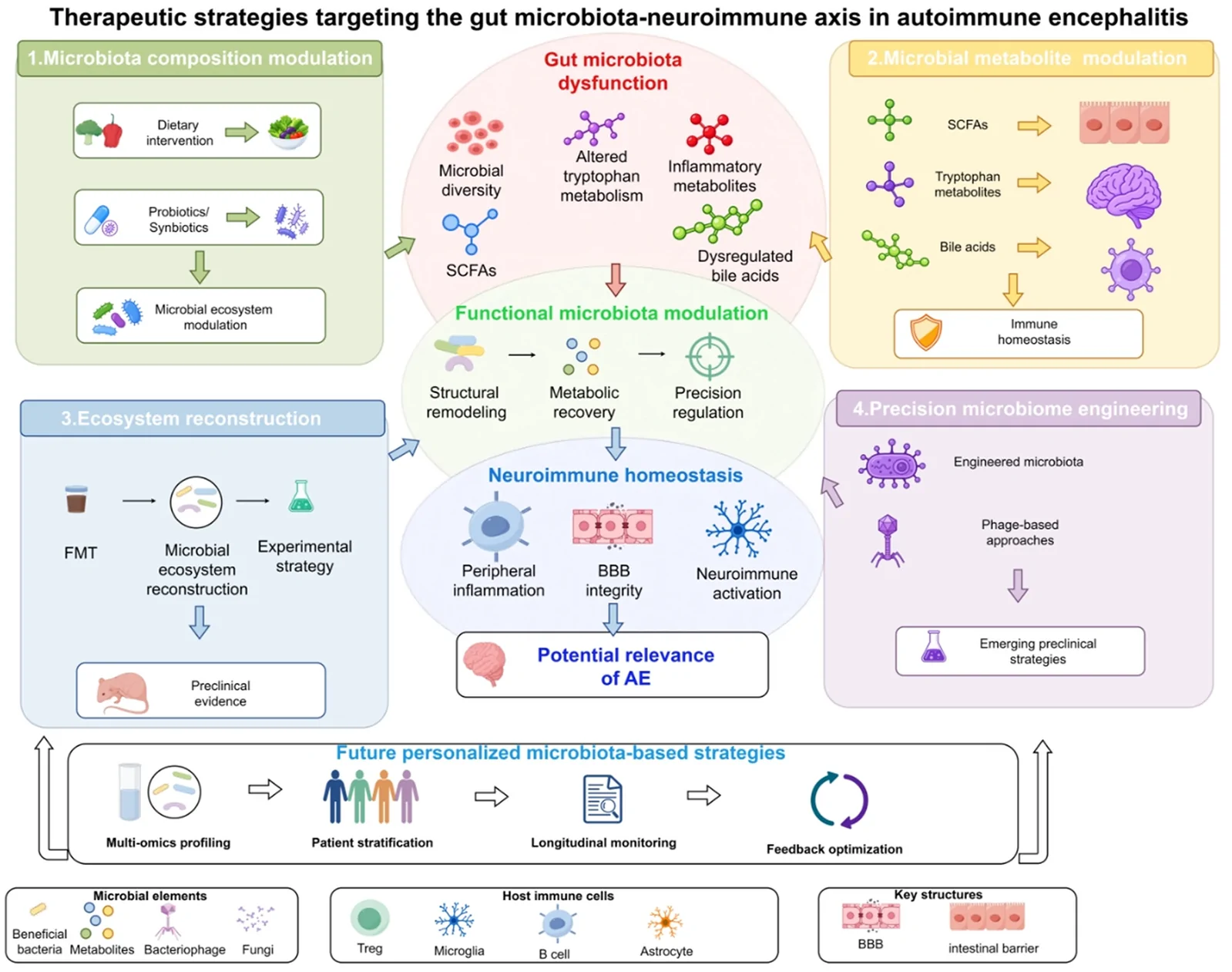
Conceptual framework of potential therapeutic strategies targeting the gut microbiota-neuroimmune axis in autoimmune encephalitis. This figure summarizes potential microbiota-targeted therapeutic strategies aimed at modulating the gut microbiota-neuroimmune axis in autoimmune encephalitis (AE). Four complementary intervention approaches are illustrated. The first involves modulation of microbial community structure, including dietary intervention, probiotics/synbiotics, and broader microbial ecosystem modulation. The second involves modulation of microbial metabolites, including strategies related to short-chain fatty acids (SCFAs), tryptophan metabolites, and bile acids. The third approach is ecosystem reconstruction, represented by fecal microbiota transplantation (FMT), which is currently experimental in AE and supported mainly by preclinical or indirect evidence. The fourth approach is precision microbiome engineering, including engineered microbiota and bacteriophage-based strategies, which remain emerging preclinical approaches. Collectively, these interventions are proposed to promote functional microbiota modulation and support neuroimmune homeostasis, with potential relevance to AE. The lower panel highlights a future personalized microbiota-based strategy integrating multi-omics profiling, patient stratification, longitudinal monitoring, and feedback optimization. This figure summarizes potential and emerging therapeutic concepts rather than established clinical treatments or treatment guidelines. SCFAs, short-chain fatty acids; FMT, fecal microbiota transplantation; BBB, blood-brain barrier.

**Table 2:** Microbiota-targeted therapeutic strategies and their potential translational value in AE.

Intervention category	Representative strategies	Major mechanisms of action	Potential relevance to AE	Evidence level/source	Major limitations	References
Dietary and nutritional interventions	High-fiber diets, fermented foods, metabolism-oriented dietary patterns	Modulate gut microbial diversity and functional capacity, promote production of SCFAs and indole-derived metabolites, and support intestinal barrier homeostasis	May reshape disease-associated microbial environments and reduce pro-inflammatory immune susceptibility relevant to AE	Indirect evidence: human dietary intervention studies and EAE mechanistic studies; no direct evidence in AE	Considerable inter-individual variability in dietary responses; optimal dietary composition and intervention window remain unclear	Shu et al., 2023; Yu et al., 2021
Probiotics/synbiotics	Lactobacillus acidophilus, *Lactobacillus rhamnosus*, and related strains combined with prebiotics	Modulate gut microbial composition and metabolic profiles, potentially improve intestinal barrier function and immune regulation	May influence peripheral inflammatory environments associated with AE; potential benefits require validation in AE-specific studies	Indirect evidence: EAE and experimental autoimmune neuritis models; AE-specific clinical evidence is lacking	Strain-specific effects, limited colonization stability, variable host responses, and uncertain long-term efficacy	Chang et al., 2023; Zrzavy et al., 2021
Modulation of SCFA pathways	Dietary fiber, propionate or butyrate supplementation, promotion of SCFA-producing bacteria	Potentially restore SCFA availability and regulate GPR43/GPR109A signaling, HDAC activity, Treg function, and immune homeostasis	May influence immune tolerance and neuroinflammatory susceptibility; relevance to AE remains hypothetical	Indirect evidence: MS clinical/mechanistic studies and EAE models; no direct intervention evidence in AE	Optimal dosage, administration route, target population, and long-term effects remain undetermined	Mosaddeghi-Heris et al., 2026; Wang H. et al., 2024
Restoration of tryptophan metabolism	Strategies enhancing indole metabolite production and modulation of the tryptophan–AhR signaling axis	Modulate AhR-dependent signaling, regulate Th17/Treg balance, and influence microglia–astrocyte interactions	May regulate immune tolerance and neuroinflammatory responses potentially involved in AE-associated immune dysregulation	Indirect evidence: EAE and experimental neuroinflammation models; no direct evidence in AE	Biological effects of AhR ligands are highly cell-type- and context-dependent	Salvo-Romero et al., 2022; Yu et al., 2021
Remodeling of bile acid metabolism	Supplementation with TUDCA/TCDCA and modulation of TGR5/GPBAR1 signaling	Modulate bile acid receptor signaling and regulate immune cell function and glial inflammatory responses	May influence neuroimmune homeostasis; potential relevance to AE requires further validation	Indirect evidence: MS metabolic studies and EAE intervention studies; no direct evidence in AE	Current evidence mainly derives from direct bile acid supplementation rather than microbiota-targeted intervention; optimal strategies remain unclear	Feng et al., 2020; Theorell et al., 2024
Regulation of lactate and other microbiota-derived small-molecule metabolites	Supplementation with lactate, indole-3-lactate, or modulation of metabolite production	Regulate NLRP3 inflammasome activity, systemic inflammatory signaling, and Th17-associated immune responses	May modify peripheral inflammatory environments relevant to neuroautoimmune disorders; AE-specific effects remain uncertain	Indirect evidence: EAE studies and non-neurological inflammatory disease models; AE relevance remains highly indirect	Limited evidence in AE; key metabolites and optimal intervention strategies have not been established	Yu et al., 2021; Taraschenko et al., 2021
Fecal microbiota transplantation (FMT)	Healthy donor microbiota transplantation and comprehensive microbial ecosystem reconstruction	Potentially modulate gut microbial composition, metabolic networks, and intestinal homeostasis, with possible effects on IL-17- and HPA axis-related signaling	May provide a strategy to investigate whether restoration of microbial ecosystems can influence immune dysregulation and neuroinflammatory susceptibility; clinical efficacy in AE remains unknown	Experimental/indirect evidence: patient-derived microbiota transfer studies and other autoimmune/neuroimmune models; no clinical therapeutic evidence in AE	Donor dependency, infection risk, heterogeneous responses, lack of standardized protocols, uncertain colonization stability, and unknown optimal timing	Taraschenko et al., 2024; Bhargava et al., 2020
Engineered bacterial therapies	Propionate-producing engineered bacteria and engineered Lactococcus/Lactobacillus strains expressing therapeutic antigens	Deliver functional metabolites or therapeutic molecules and enable targeted modulation of microbial functions	May provide future precision microbiome-based interventions; application in AE remains unexplored	Indirect evidence: EAE and experimental intestinal inflammation models; no translational evidence in AE	Biosafety concerns, genetic stability, colonization control, and regulatory challenges	Xu et al., 2023; Li et al., 2022
Bacteriophage therapy	Phages or phage cocktails targeting pro-inflammatory commensal or opportunistic pathogenic bacteria	Selectively modulate specific bacterial populations while preserving broader microbial community structure	May enable strain-level precision remodeling of gut microbiota; potential value in AE remains hypothetical	Indirect evidence: animal studies in inflammatory diseases; no evidence in AE or neuroautoimmune diseases	Identification of AE-relevant target strains, narrow host range, and phage resistance remain major challenges	Wang et al., 2021; Xu et al., 2024

### 6.1 Strategies for modulating microbial community structure

Alterations in gut microbial composition represent one of the major observations reported in microbiota studies of AE, raising the possibility that modulation of microbial community structure may have therapeutic relevance. Dietary modification, probiotics, and synbiotics may alter the intestinal ecological environment, reshape microbial community composition, and influence microbial functional outputs.

Dietary intervention represents one of the most accessible approaches to modulating the gut microbiota. High-fiber diets and dietary patterns rich in plant-derived components can promote the growth of metabolically beneficial commensal microorganisms and influence microbial metabolic capacity. However, AE-associated microbial alterations may themselves be affected by age, dietary habits, medication exposure, disease severity, and treatment status. The effects of nutritional interventions on microbial composition, immune phenotypes, and clinical outcomes therefore require direct evaluation in patients with AE.

Probiotics and synbiotics aim to modify selected microbial functions by introducing microorganisms with potentially beneficial properties or by promoting their growth and activity. AE-associated dysbiosis, however, does not appear to reflect the expansion of a single pathogenic microorganism and is more likely to involve complex ecological and functional alterations. Supplementation with an individual bacterial strain may therefore be insufficient to address such disturbances. Future probiotic strategies may increasingly focus on modulation or supplementation of specific microbial functions, potentially through microbial consortia selected according to patient-specific alterations in microbial composition and metabolic capacity.

### 6.2 Strategies for modulating microbial metabolic function

Compared with modification of microbial composition alone, targeting microbial metabolic function may more closely reflect the proposed mechanisms through which the gut microbiota influences immune regulation. Microbiota-derived metabolites may affect immune homeostasis, barrier integrity, and the neuroinflammatory environment, making microbial metabolic pathways potential targets for future intervention.

SCFAs are among the most extensively studied functional metabolites of the gut microbiota. AE-related microbiota-transfer studies suggest that patient-derived microbial communities may be associated with reduced SCFA production, impaired BBB stability, and enhanced inflammatory responses. However, most evidence supporting SCFA supplementation or modulation of SCFA-producing microbial communities derives from MS, EAE, and other autoimmune disease models. Whether such strategies provide therapeutic benefit in AE has not been directly demonstrated.

Beyond SCFAs, tryptophan and bile acid metabolism represent additional candidate pathways for functional modulation. Microbiota-derived tryptophan metabolites can influence immune-cell function through AhR-dependent signaling, whereas microbially transformed bile acids can regulate the Th17/Treg balance and immune tolerance. At present, however, these mechanisms have been defined primarily in related neuroimmune and inflammatory disease models. Whether comparable metabolic abnormalities occur in AE, whether antibody-defined subtypes exhibit distinct metabolic signatures, and which disease phases might be most appropriate for targeting these pathways remain important questions for future investigation.

### 6.3 Whole-ecosystem reconstruction: fecal microbiota transplantation

Because AE-associated dysbiosis may involve multiple microbial taxa and complex metabolic networks, interventions directed at a single strain may be insufficient to modify the broader disease-associated microbial state. Fecal microbiota transplantation (FMT), which introduces a complex microbial community together with its associated functional potential, provides an experimental approach for investigating whether coordinated modulation of microbial structure and function can influence neuroimmune homeostasis.

In principle, FMT may modify microbial diversity, metabolic networks, and ecological stability. However, these potential effects are inferred largely from general principles of microbiome modulation and studies in other disease settings. FMT cannot currently be regarded as an intervention proven to improve clinical outcomes in patients with AE. Its principal value in AE at present lies in exploring whether broad manipulation of the microbial ecosystem can influence immune homeostasis, inflammatory signaling, and neuroimmune processes rather than in its use as an established therapeutic modality.

To date, no clinical studies have directly evaluated the safety or therapeutic efficacy of FMT in AE. Experiments involving transplantation of microbiota derived from patients with AE suggest that disease-associated microbial communities may influence Th17-related immune responses, the metabolic environment, and BBB function, thereby providing mechanistic evidence for microbiota–neuroimmune interactions. Patient-derived microbiota-transfer experiments in AE support the biological relevance of disease-associated microbial communities, whereas FMT studies in other disease models suggest that broad ecosystem manipulation can modify microbial and immune states. However, direct evidence of clinical benefit from FMT in AE is lacking. Similarly, FMT studies in other neuroimmune and autoimmune disease models provide only indirect experimental evidence, and their relevance to AE remains uncertain.

Several limitations currently constrain the potential application of FMT in AE. First, it remains unclear whether AE is associated with a stable and disease-specific pattern of dysbiosis, and different antibody-defined subtypes, disease phases, and treatment states may be associated with distinct microbial profiles. Second, because FMT introduces highly complex microbial communities, donor selection, long-term safety, durability of microbial engraftment, and optimal timing of intervention require systematic evaluation. A recent review of FMT in neurological disorders highlighted its potential relevance to modulation of the gut–brain axis while emphasizing major translational barriers, including donor screening, stable microbial engraftment, long-term safety, and disease-specific variability in treatment response (Eslami et al., 2025). Accordingly, FMT should currently be regarded as an experimental and investigational microbiota-directed strategy in AE. Prospective studies integrating microbial profiles, metabolic states, and immune phenotypes will be required to determine whether it has meaningful translational value.

### 6.4 Precision microbiome engineering

Advances in metagenomics and synthetic biology have created opportunities to move from broad ecological modulation toward more precise manipulation of microbial functions. Engineered microorganisms and bacteriophage-based approaches represent two emerging strategies within this framework. Their aim is not simply to alter microbial abundance but to selectively modify defined functions or microbial populations within the intestinal ecosystem.

Engineered bacterial platforms can be designed to perform specific metabolic functions, including the controlled production or delivery of selected metabolites. In neuroimmune disorders characterized by reduced SCFA availability or altered immunomodulatory metabolism, engineered strains could theoretically provide a more targeted and controllable means of modifying microbial functional outputs. At present, however, relevant evidence is derived primarily from EAE and other inflammatory disease models, and the applicability of engineered microbial therapeutics to AE remains theoretical.

Bacteriophage-based approaches offer the possibility of strain-level precision in microbial modulation. Unlike broad-spectrum antimicrobial strategies, bacteriophages can selectively target specific bacterial populations while potentially limiting disruption of the surrounding microbial community. However, microbial taxa with a demonstrated causal role in AE pathophysiology have not yet been identified. Phage-based intervention in AE therefore remains at the proof-of-concept stage. Future precision microbiome approaches will likely require integration of metagenomic, metabolomic, and immune-phenotypic data to identify patient-specific microbial functional abnormalities and determine whether they represent actionable therapeutic targets.

Importantly, microbiota-directed strategies currently occupy substantially different stages of development, and the evidence supporting them should not be considered equivalent. Dietary interventions, probiotics/synbiotics, and selected strategies targeting microbial metabolism are supported primarily by mechanistic studies, animal experiments, and limited human observational evidence. Patient-derived microbiota-transfer experiments in AE support the biological relevance of disease-associated microbial communities, whereas FMT studies in other disease models suggest that broad ecosystem manipulation can modify microbial and immune states. However, direct evidence of clinical benefit from FMT in AE is lacking. Engineered microorganisms and bacteriophage-based approaches remain largely preclinical or proof-of-concept, and their application to AE is currently theoretical. At present, no gut microbiota-targeted intervention has been demonstrated to improve clinical outcomes in patients with AE.

Overall, microbiota-directed intervention is evolving from broad manipulation of microbial composition toward increasingly precise modulation of microbial function. Dietary interventions, probiotics, and FMT primarily target microbial community structure and ecological organization, whereas strategies involving SCFAs, tryptophan metabolism, and bile acid metabolism focus more directly on microbial functional outputs. Precision microbiome engineering may eventually provide more individualized approaches to functional modulation. Nevertheless, current evidence in AE remains predominantly mechanistic and preclinical, and clinical studies establishing safety and efficacy are lacking. Future research should integrate microbial signatures, metabolic functions, neuroimmune phenotypes, and clinical outcomes within longitudinal cohorts and carefully designed interventional studies to determine the actual translational value of microbiota-targeted strategies in AE.

## 7 Future perspectives

Bidirectional interactions between the gut microbiota and the neuroimmune system have emerged as a useful conceptual framework for exploring the pathophysiology of AE. Current evidence clearly supports the presence of immune dysregulation in patients with AE and further suggests that alterations in the gut microbiota may be associated with host immune dysregulation and neuroinflammatory processes. However, the available evidence is derived predominantly from observational studies in patients, animal experiments, and mechanistic studies in related neuroimmune disorders. It therefore remains unclear whether gut microbial alterations directly contribute to AE initiation or progression, whether reproducible disease-specific microbial signatures exist, and how distinct microbiota-derived signals vary across the disease course.

Future research should move beyond descriptive analyses of microbial composition toward characterization of microbial functions, metabolic networks, and their dynamic relationships with host immune phenotypes. Longitudinal sampling across different disease phases, combined with metagenomics, metabolomics, immune profiling, and functional validation, will be particularly important for distinguishing disease-associated microbial changes from treatment-related or secondary alterations. Such approaches may help identify microbial signals and functional pathways that are consistently associated with AE and clarify whether any of these represent biologically or clinically actionable targets.

From a translational perspective, gut microbiota–neuroimmune crosstalk provides a potential framework for the development of microbiota-directed strategies in AE. Nevertheless, these approaches remain largely at the mechanistic, experimental, or preclinical stages, and no microbiota-targeted intervention has yet been demonstrated to improve clinical outcomes in patients with AE. Dietary modulation, probiotics, modulation of microbial metabolic functions, FMT, and precision microbiome engineering all warrant further investigation, but their efficacy, safety, optimal timing, and appropriate target populations require rigorous clinical evaluation.

Looking forward, integration of single-cell technologies, multi-omics profiling, functional microbiology, and longitudinal clinical phenotyping may enable a more comprehensive understanding of the relationships among microbial alterations, metabolic signaling, immune remodeling, barrier dysfunction, and neuronal injury. Importantly, future studies should also incorporate AE subtype, disease phase, treatment exposure, and relevant host factors into study design and analysis. Such work will be essential for determining which components of the gut microbiota–neuroimmune axis represent reproducible disease-associated features, which may have mechanistic relevance, and which, if any, can ultimately support precision diagnostic or therapeutic strategies in AE.
